# Individual-Level Socioeconomic Position and Long-Term Prognosis in Danish Heart-Transplant Recipients

**DOI:** 10.3389/ti.2023.10976

**Published:** 2023-03-22

**Authors:** Rikke E. Mols, Brian B. Løgstrup, István Bakos, Erzsébet Horváth-Puhó, Bo Christensen, Christoffer T. Witt, Morten Schmidt, Finn Gustafsson, Hans Eiskjær

**Affiliations:** ^1^ Department of Cardiology, Aarhus University Hospital, Aarhus, Denmark; ^2^ Department of Clinical Medicine, Aarhus University, Aarhus, Denmark; ^3^ Department of Clinical Epidemiology, Aarhus University Hospital, Aarhus University, Aarhus, Denmark; ^4^ Department of Public Health, Research Unit for General Medicine, Aarhus University, Aarhus, Denmark; ^5^ Department of Cardiology, University Hospital of Copenhagen, Copenhagen, Denmark; ^6^ Department of Clinical Medicine, University of Copenhagen, Copenhagen, Denmark

**Keywords:** mortality, heart transplantation, prognosis, individual-level, socioeconomic position

## Abstract

Socioeconomic deprivation can limit access to healthcare. Important gaps persist in the understanding of how individual indicators of socioeconomic disadvantage may affect clinical outcomes after heart transplantation. We sought to examine the impact of individual-level socioeconomic position (SEP) on prognosis of heart-transplant recipients**.** A population-based study including all Danish first-time heart-transplant recipients (*n* = 649) was conducted. Data were linked across complete national health registers. Associations were evaluated between SEP and all-cause mortality and first-time major adverse cardiovascular event (MACE) during follow-up periods. The half-time survival was 15.6 years (20-year period). In total, 330 (51%) of recipients experienced a first-time cardiovascular event and the most frequent was graft failure (42%). Both acute myocardial infarction and cardiac arrest occurred in ≤5 of recipients. Low educational level was associated with increased all-cause mortality 10–20 years post-transplant (adjusted hazard ratio [HR] 1.95, 95% confidence interval [CI] 1.19–3.19). During 1–10 years post-transplant, low educational level (adjusted HR 1.66, 95% CI 1.14–2.43) and low income (adjusted HR 1.81, 95% CI 1.02–3.22) were associated with a first-time MACE. In a country with free access to multidisciplinary team management, low levels of education and income were associated with a poorer prognosis after heart transplantation.

## Graphical Abstract

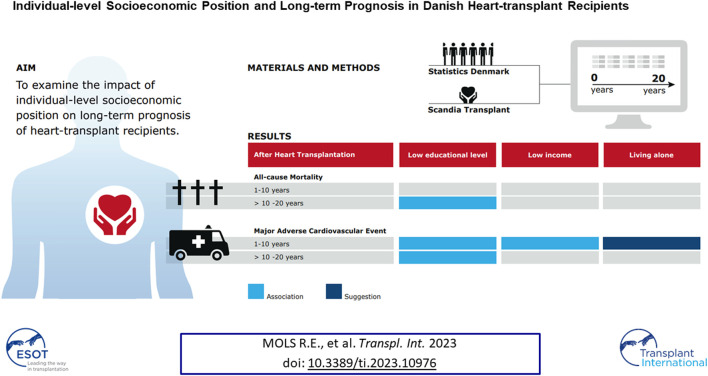

## Introduction

Heart transplantation is a widely accepted procedure improving survival, quality of life, and physical capacity in patients with end-stage heart failure (1, 2). During the past 30 years, survival rates have increased significantly, despite high-risk and older recipients undergoing heart transplantation (1, 3). Currently, the 50% survival estimate after heart transplantation in adults is 12.5 years, and 14.8 years when conditional on 1-year survival (3). Advances in immunosuppressive treatment and perioperative care have improved 1-year survival to approximately 90% (4). The main causes of death immediately following heart transplantation are primary graft dysfunction, rejection, and infection; primary causes of long-term mortality are cardiac allograft vasculopathy, non-specific graft failure, renal dysfunction, and malignancy (3, 4). It is pivotal for follow-up of heart transplant recipients that transplant centers establish multidisciplinary team management programs, designed to improve survival (2, 5).

Studies in both the United States and the United Kingdom have shown that multiple indicators of index-based socioeconomic position (SEP) are associated with death, independent of baseline clinical characteristics of heart transplant recipients (6–8). Among American heart transplant survivors, low SEP (score) predicted an increased risk of rejection and graft loss (9). Earlier studies in the United States have suggested higher mortality in patients covered by Medicare compared with patients covered by private insurance at the time of heart transplantation (6, 10). Studies primarily conducted in the United States have also reported that depression before or early after heart transplantation is associated with higher post-transplant mortality (11–13). Mental health conditions often coexist with physical chronic diseases (14). Multimorbidity including chronic psychiatric disorder has been associated with higher mortality (14, 15). Moreover, data support a strong socioeconomic gradient in the onset of multimorbidity (16, 17). However, important gaps persist in the understanding of how individual indicators of socioeconomic deprivation and comorbidities affect clinical outcomes after heart transplantation in European universal healthcare systems with free access to multidisciplinary team management programs.

The structure and content of Danish healthcare registers ensure a unique and virtually complete individual-level linkage of data and long-term follow-up (18). Furthermore, the universal healthcare model in Denmark provides health service free of charge to all residents. We used the Scandiatransplant Database (STD) and nationwide health and administrative registers to examine the 20-year prognosis of all heart-transplant recipients in Denmark and the prognostic impact of individual-level SEP and comorbidities.

## Matrials and Methods

### Setting

The Danish national healthcare system provides tax-financed healthcare for all residents at general practitioners and hospitals as well as reimbursement of prescribed medical therapy. The Civil Registration System (CRS) can unambiguously link up-to-date national health and administrative register data using a unique 10-digit identifier assigned to all residents at birth or upon immigration (18). Denmark has two transplant centers at the University Hospital of Copenhagen and at Aarhus University Hospital.

### Data Sources

This study was based on data from: 1) STD, which covers data on all Danish heart-transplant recipients and donors (19), 2) The Danish National Patient Registry (DNPR) (18) containing information on discharge diagnosis according the International Classification of Diseases (ICD-8 and since 1994 ICD-10 codes), along with codes for diagnostic and surgical procedures (18), 3) The Psychiatric Central Research Register (PCRR) containing information on psychiatric diagnoses (18), 4) The Danish National Prescription Registry (NPR) (18), containing data on all redeemed prescriptions at Danish community pharmacies (18). Medical therapies were identified by substance level (Anatomical Therapeutic Chemical [ATC] Classification), 5) The Danish Causes of Death Registry (DCDR) (18), where causes of death are listed as the immediate, underlying, and contributing cause of death (18), 6) CRS including data on vital status, date of birth, gender, and marital status (18), and finally 7) Statistics Denmark (18) covering information from the Education Registry, the Income Statistics Register, and the Integrated Database for Labor Market Research.

This study was approved by the Danish Data Protection Agency (no: 1-16-02-656-18) and the Danish Patient Safety Authority, authorizing access to medical records (no: 3-3013-3173/1).

### Study Population and Characteristics

We established a nationwide cohort study including Danish first-time heart-transplant recipients during 1994–2018 recorded in the STD by ICD-10 code (DZ94.1). The index date was defined as the date of the first surgical heart transplantation in the STD. Heart-transplant recipients were followed from index date until 31 December 2018, emigration, or death, whichever occurred first. Recipients undergoing re-transplantation identified in the DNPR (KFQA00, KFQA10) were not censored, since reoperation would be part of the causal pathway of long-term outcome. Information on age, gender, and vital status was retrieved from the CRS (18). Data on donor age and gender mismatch (donor/recipient) were extracted from the SDT.

Age at index date was categorized as 0-20, 21-40, 41-60, and ≥61 years, due to increasing complexities in early, middle, and long-term management post-surgery (20); follow-up time was defined as 0–1, >1–10, and >10 years. The number of recipients alive at end of follow-up was calculated. We collected information on clinically relevant comorbidities by ICD codes registered in the DNPR (18) and PCRR (18) 10 years prior to the index date: Myocardial infarction, angina pectoris, heart failure, heart valve disease, cardiac arrhythmia, congenital heart disease, cardiomyopathy, cardiac inflammation, aortic disease, peripheral arterial disease, cerebrovascular disease, cardiogenic shock and pulmonary edema, diabetes, hypertension, chronic obstructive pulmonary disease, obesity, and psychiatric disorder (Supplementary Table S1). Based on the definition of multimorbidity in other Danish studies (20, 21), we summarized the number of comorbidities 10 years prior to the index date. This Danish algorithm estimates multimorbidity as the co-occurrence of two or more chronic conditions included in the 11 comprehensive chronic disease groups (Supplementary Table S3). Medical treatment was defined as ≥1 redeemed prescription 6 months prior to the index date retrieved from the NPR (18). Polypharmacy was defined as redeeming at least one prescription for ≥5 different cardiovascular agents (18) (Supplementary Table S3).

### Individual-Level Socioeconomic Position

Data on individual-level SEP were obtained from Statistics Denmark. Cohabitation status at index date was defined as living alone or cohabiting. We used the highest attained educational level in the calendar year prior to the index date (18) and categorized educational level into five groups: Low (primary and lower secondary education), medium (upper secondary education and academy profession degree), high (bachelor and above), not completed an education (patients age ≤16 years), and missing. We used personal income (pre-tax total) within the calendar year prior to the index date. Based on the annual percentiles in the Danish population, we classified income into percentiles and used the 25th percentile as the cut-off point for low (≤25th percentile) and medium-high (>25th percentile) personal income. Occupational status in the year prior to the index date (18) was grouped into working, non-working (no employment or early retirement), out-of-workforce (state pension, under education), and missing (Supplementary Table S4).

### Outcomes

We used the CRS (18) to ascertain date on all-cause mortality during the years following the index date. We also examined cause of mortality using information from the DCDR (18). Cause of mortality was defined by underlying cause and possible cause (immediate cause when available, 1st contributory cause when immediate cause was missing, or 2nd contributory cause when immediate and 1st contributory cause was missing). We generated a list of all documented causes (ICD-10 codes) and divided these into twelve categories: Complications to heart transplantation, multiple organ failure, sudden death, cardiovascular disease, heart failure, cerebrovascular disease, infection, pulmonary disease, malignancy, kidney disease, diabetes, other specified, and not specified (Supplementary Table S5).

The first-time occurrence of hospital admission with a cardiovascular event after the index date was examined (acute myocardial infarction, peripheral arterial disease, cardia arrest, stroke, cardiac inflammation and infection, readmission due to heart failure, graft failure, percutaneous coronary intervention, radiofrequency ablation for atrial fibrillation, cardiac pacemaker, and valve surgery) (Supplementary Table S6). Information was retrieved from the DNPR by primary in-patient diagnosis and surgical procedure codes (18). We investigated the risk of first-time major adverse cardiovascular event (MACE). Composite MACE included readmission due to heart failure, graft failure, percutaneous coronary intervention, acute myocardial infarction, cardiac arrest, and all-cause mortality. To account for potential misclassification of first-time occurrence of hospital admission due to a MACE (especially graft failure due to standard biopsy controls in the first post-transplant year; heart failure, which could follow from prior index date), we conducted a blanking period of 365 days after the index date (Supplementary Table S7).

### Statistical Analyses

Baseline data were reported as mean and standard deviation (SD) if normally distributed and as median with 25th‒75th interquartile range (IQR) if skewed continuous data. Categorical data were presented as prevalence (percentage).

Cause of mortality and first-time cardiovascular events were recorded in numbers and percentages. The Kaplan-Meier method was used to compute the risk of all-cause mortality (All). Conditional analyses were performed in recipients who survived the first year (1-year Post-surgery Survival). As supplementary, survival was stratified by time era (1994–2000, 2001–2010, 2011–2018). In addition, the Kaplan-Meier method was used to compute the risk of first-time MACE using the first year after the index date as a blanking period (1-year Post-surgery MACE). Due to Danish law on data protection, first-time acute myocardial infarction (≤5) and cardiac arrest (≤5) were not included in the MACE. However, sensitivity analysis including these events did not change the results. As supplementary, survival and first-time MACE were stratified by gender. To identify the most socially disadvantaged recipients, all socioeconomic factors were dichotomized by the worst quartile or lowest status. Recipients with low educational level (low-degree) were compared to those with medium-higher educational level (medium-high-degree). Recipients <16 years and with missing information on education were not included. Prognostic outcomes were assessed among unemployed (non-working) compared to employed (working, out-of-workforce) recipients. In case of missing information on occupational status, recipients were excluded. Due to the limited sample size, it was not possible to further categorize the exposure variables.

Based on the increasing complexity in long-term management after transplantation (20), we determined the impact of all exposure variables on prognostic outcomes within follow-up intervals: 0–1, >1–10, and >10–20 years. Crude and adjusted hazard ratios (HRs) were computed using Cox Proportional Hazards regression comparing recipients within the dichotomized socioeconomic groups. In multivariable analyses, we adjusted for age, gender, donor age, gender mismatch, hypertension, and diabetes. We evaluated the proportional hazards assumption by visual inspection of log-log plots. Since the median number of comorbidities at baseline was one and less than 2% of the recipients had a psychiatric disorder, these two covariates did not change the results and were thus not included in the regression. We found no indication of any difference between the two Danish transplant centers and transplantation site was not distinguished between in the analyses. A *post hoc* power analysis was not performed as the utility to inform outcome already observed seems analytically misleading (22). All statistical analyses were performed using SAS statistical software package (version 9.4) and R version 4.1.0 (2021-05-18).

## Results

Between 1994 and 2018, first-time heart transplantation was performed in 649 recipients in Denmark (Table 1). Most recipients were male (78%) and 59% were between 41 and 60 years of age at surgery date. Diabetes and hypertension both occurred in 12% of recipients. The median (IQR) number of comorbidities within 10 years prior to transplantation was 1 (1–2). Psychiatric disorder was present in ≤5 of recipients. Median donor age was 41 (IQR, 27–50) and gender mismatch was present in 29%.

**TABLE 1:** Baseline characteristic in heart-transplant recipients.

	Total
	N = 649
Gender	
Male	503 (78)
Female	146 (22)
Age in years	
0–20	67 (10)
21–40	117 (18)
41–60	381 (59)
≥61	84 (13)
Donor	
Age, median (IQR)	41 (27–50)
Gender mismatch	118 (29)
Follow-up time in years	
0–1	97 (15)
>1–10	296 (46)
>10	256 (39)
Median (IQR)	7.4 (2.7–13.7)
Alive at end of follow-up	375 (58)
Comorbidities (10 years prior to the index date)	
Myocardial infarction	211 (33)
Angina Pectoris	272 (42)
Heart failure	547 (84)
Heart valve disease	71 (11)
Cardiac arrhythmia	307 (47)
Congenital heart disease	70 (11)
Cardiomyopathy	434 (67)
Cardiac inflammation	66 (10)
Aortic disease	**—** ^a^
Peripheral arterial disease	10 (2)
Cerebrovascular disease	61 (9)
Cardiogenic shock and pulmonary edema	57 (9)
Diabetes	77 (12)
Hypertension	80 (12)
Chronic obstructive pulmonary disease	69 (11)
Obesity	21 (3)
Mental disease	**—** ^a^
Multimorbidity (10 years prior to the index date)	
Number of chronic diseases, median (IQR)	1 (1–2)
Cardiovascular polypharmacy (6 months prior to the index date) ^b^	
Prescribed medications ≥5	348 (54)
Cohabitation status	
Living alone	281 (43)
Cohabitation	368 (57)
Highest obtained educational degree	
Low (primary and lower secondary education)	193 (30)
Medium (upper secondary education and academy profession)	283 (44)
High (bachelor and above)	116 (18)
Not completed education (patients age ≤16 years)	40 (6)
Missing	17 (3)
Personal income group	
Low income (≤25th percentile)	134 (21)
Medium-high income (>25th percentile)	515 (79)
Occupational status	
Working	300 (46)
Non-working	27 (4)
Out-of- workforce (state pension, under education)	300 (46)
Missing	22 (3)

Values are n (%).

^a^ Due to data protection (<5 patients).

^b^ Data available since 1995 in the Danish National Prescription Registry.

### Outcomes

**FIGURE 1:**
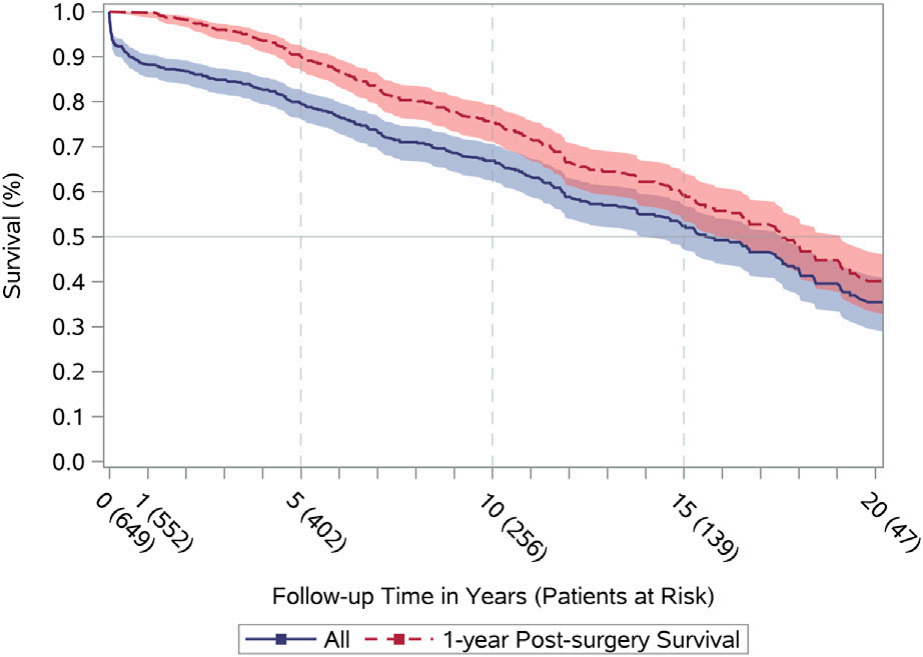
Long-term survival. All (blue), All-cause mortality after surgery date (index date). 1-year Post-surgery Survival (red), Conditional all-cause mortality in recipients who survived first year after the index date.

**FIGURE 2:**
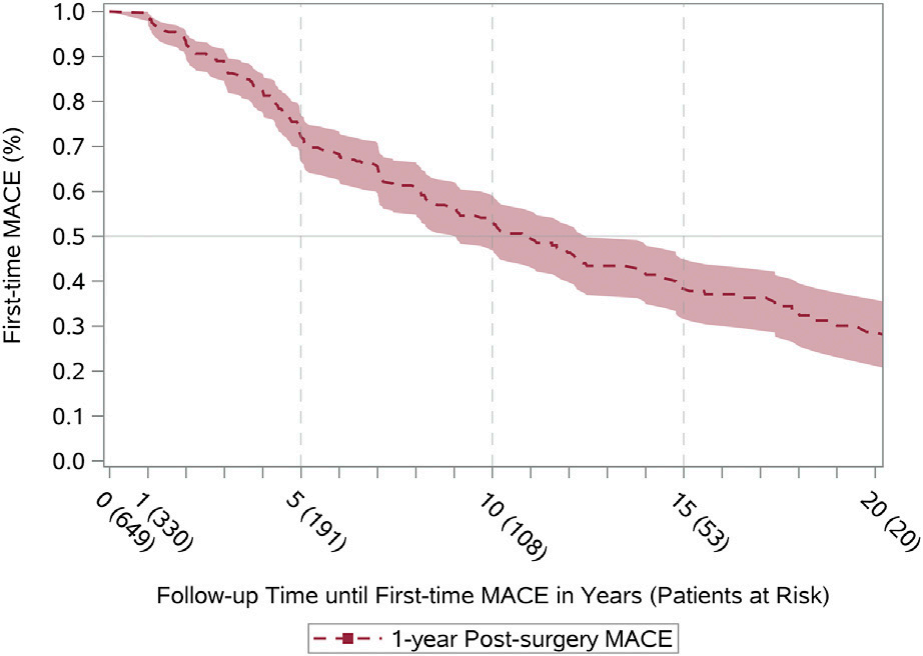
Long-term first-time MACE. Conditional first-time MACE in recipients who survived first year after the index date (1-year Post-surgery MACE). MACE, Major Adverse Cardiovascular Event (composite of readmission due to heart failure, graft failure, percutaneous coronary intervention, and all-cause mortality).

Twenty-year survival curves for all-cause mortality are displayed in Figure 1. The half-time survival was 15.6 years (95% confidence interval [CI] 13.8–17.5) and 17.6 years when conditional on 1-year survival (95% CI 16.2–19.1) (Supplementary Figures S1–S4). The leading underlying causes of mortality were heart failure (25%), cardiovascular disease (18%), and malignancy (18%) (Table 2). The three cardiovascular first-time events with the highest incidence (within 1–20 years post-surgery) were graft failure (42%), readmission due to heart failure (14%), and percutaneous coronary intervention (21%) (Table 3). Both acute myocardial infarction and cardiac arrest occurred in ≤5 of recipients. Approximately half of the heart transplant recipients were at risk of a first-time MACE within an 11-year period after the index date among those surviving to at least 1-year (Figure 2).

**TABLE 2:** Cause of mortality in heart-transplant recipients.

Overall (N = 649)	Underlying cause	Possible cause^a^
	*n* = 274	*n* = 274
Complications of heart transplantation	**—** ^b^	16 (6)
Multiple organ failure	**—** ^b^	19 (7)
Sudden deaths	**—** ^b^	25 (9)
Cardiovascular disease	48 (18)	18 (7)
Heart failure	68 (25)	32 (12)
Cerebrovascular disease	**—** ^b^	15 (5)
Infection	12 (4)	18 (7)
Pulmonary disease	**—** ^b^	23 (8)
Malignancy	48 (18)	22 (8)
Kidney disease	**—** ^b^	12 (4)
Diabetes	**—** ^b^	**—** ^b^
Other specified	**—** ^b^	**—** ^b^
Not specified	55 (20)	39 (14)

Recipients were followed after heart transplantation (index day) and until 31 December 2018, emigration, or mortality, whichever occurred first.

Values are n (%).

^a^ Immediate cause when it is available; 1st contributory cause when immediate cause is missing; 2nd contributory cause when immediate and 1st contributory cause is missing.

^b^ Due to data protection (<5).

**TABLE 3:** First-time cardiovascular event in heart-transplant recipients.

Overall (N = 649)	*n* = 330
Acute myocardial infarction	**—** ^a^
Peripheral arterial disease	11 (3)
Cardiac arrest	**—** ^a^
Stroke	10 (3)
Cardiac inflammation and infection	**—** ^a^
Readmission due to heart failure	47 (14)
Graft failure	140 (42)
Percutaneous Coronary Intervention	68 (21)
Radiofrequency ablation for atrial fibrillation	**—** ^a^
Cardiac pacemaker	18 (6)
Valve surgery	11 (3)

Recipients were followed from day +365 after heart transplantation (index day) and until 31 December 2018, emigration, or death, whichever occurred first.

Values are n (%).

^a^ Due to data protection (<5 events).

### Individual-Level Socioeconomic Position

**FIGURE 3:**
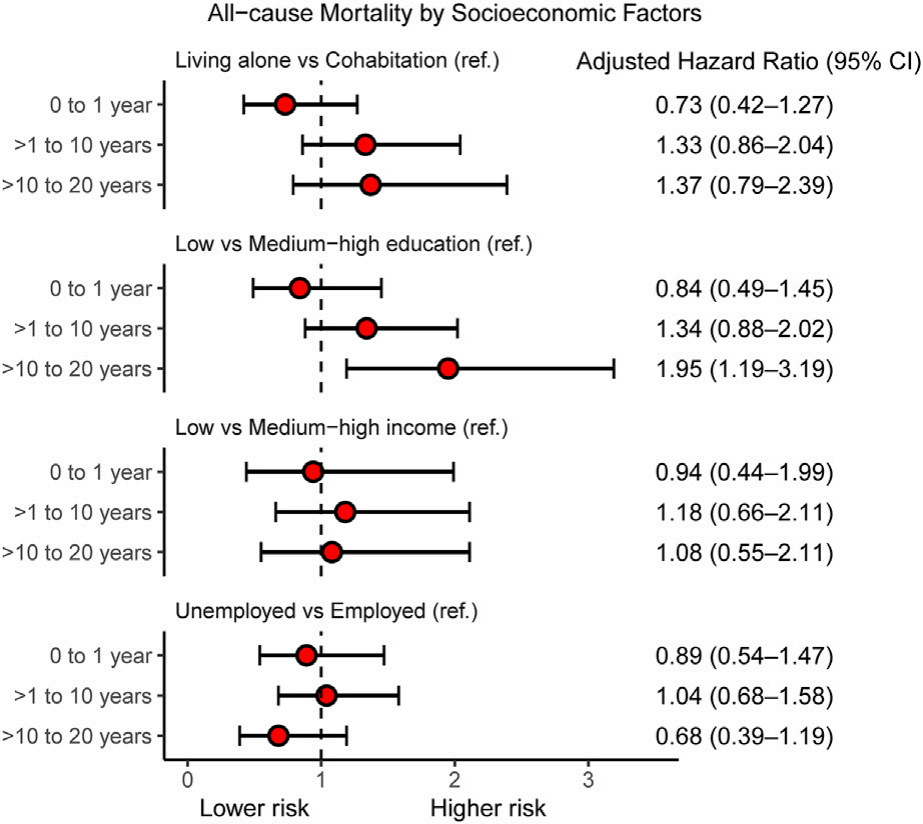
Individual-level socioeconomic position and all-cause mortality. Cox Proportional Hazard models for adjusted hazard ratios for all-cause mortality within follow-up intervals: 0–1 year, >1–10 years, and >10–20 years after heart transplantation in Denmark (1994–2018) according to socioeconomic factors. In multivariate analyses, the hazard ratios are adjusted for age, gender, donor age, gender mismatch, hypertension, and diabetes. CI, confidence interval.

**FIGURE 4:**
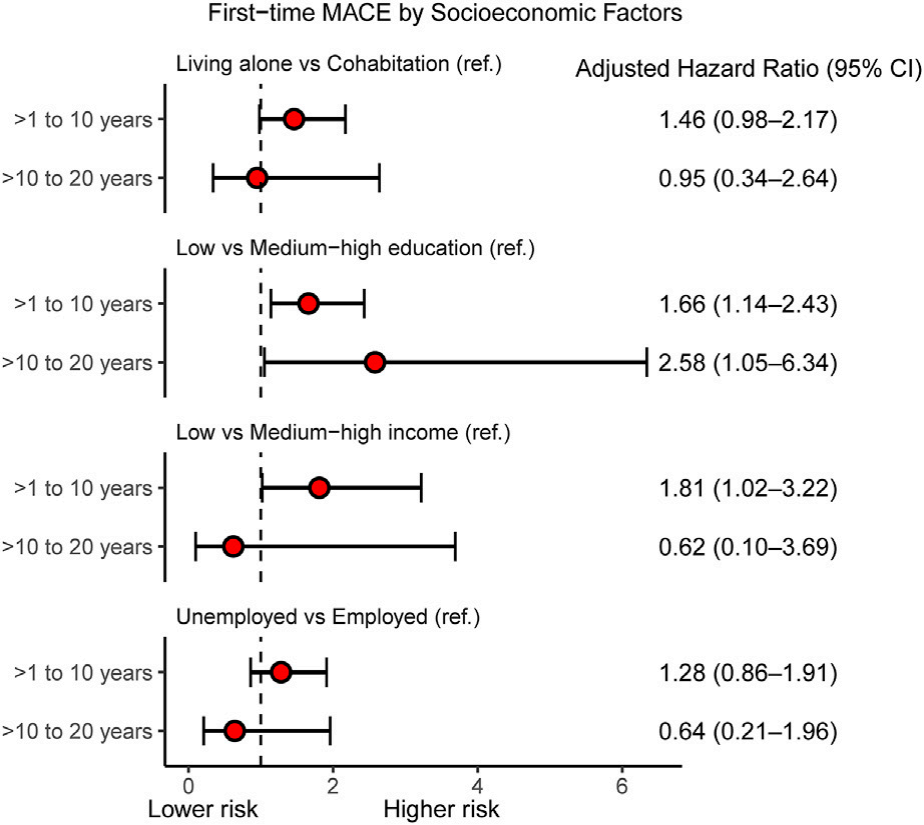
Individual-level socioeconomic position and first-time MACE. Cox Proportional Hazard models for adjusted hazard ratios for first-time MACE within follow-up intervals: >1–10 years and >10–20 years after heart transplantation according to socioeconomic factors. In multivariate analyses, the hazard ratios are adjusted for age, gender, donor age, gender mismatch, hypertension, and diabetes. MACE, Major Adverse Cardiovascular Event (composite of readmission due to heart failure, graft failure, percutaneous coronary intervention, and all-cause mortality); CI, confidence interval.

Adjusted HRs for all-cause mortality by socioeconomic factors and in different follow-up intervals are presented in Figure 3. Low educational level was associated with all-cause mortality within the period 10–20 years after heart transplantation (HR 1.95, 95% CI 1.19–3.19); otherwise we found no associations between socioeconomic factors and all-cause mortality (Supplementary Table S8). In contrast, we observed SEP-related associations with first-time MACE (Figure 4). During both >1–10 years and >10–20 years after the index date, low educational level was associated with first-time MACE. Low income was associated with first-time MACE within >1–10 years after the index date (HR 1.81, 95% CI 1.02–3.22). Cohabitation status was not significantly associated with first-time MACE during follow-up intervals. However, although it did not reach significance there was a suggestion that living alone was associated with a higher risk of first-time MACE within >1–10 years (HR 1.46, 95% CI 0.98–2.17). No associations between occupational status and first-time MACE were documented (Figure 4) (Supplementary Table S9).

## Discussion

In this nationwide cohort study comprising all Danish first-time heart-transplant recipients during a 20-year period, the half-time survival estimate was 15.6 years. The highest prevalence of first-time cardiovascular events was graft failure. This study revealed two major findings: 1) low educational level at index date was associated with higher risk of all-cause mortality within 10–20 years after heart transplantation and 2) low educational level, low income, and a suggestion towards living alone were associated with higher risk of first-time MACE within 1–10 years post-transplant.

In a Scandinavian cohort (1983–2009) of heart-transplant recipients (*n* = 2293; 8% <18 years), the half-time survival was estimated to 13.2 years (19) and 15.3 years when conditional survival was set at 1-year. We demonstrated an excellent half-time survival (15.6 years) as well as 1-year conditional survival (17.6) when compared with internationally published data. This may be attributed to heart-transplant survival consistently improving over the last 30 years and has been described as the era effect. Heart transplantation in Denmark was initiated later than in the United States and other European countries (3, 19, 23). This is supported by our supplementary survival curves in Danish heart-transplant recipients stratified by time period (1994–2000, 2001–2010, 2011–2018). In the current study, we found that the three cardiovascular first-time events with the highest incidence were graft failure, readmission due to heart failure, and percutaneous coronary intervention. Approximately half of the heart-transplant recipients were at risk of a first-time MACE within 11 years after transplantation conditional on survival of at least 1-year. Our findings consolidate that graft failure and rejection remain the leading causes of mortality post-transplant (3, 4). Cardiac allograft vasculopathy is the main reason for allograft failure, and percutaneous coronary intervention is usually considered a palliative treatment because of the progressive nature of vasculopathy (24). In addition, a recent study based on the nationwide readmission database in the United States reported that heart failure is one of the main primary unplanned diagnoses causing readmission after heart transplantation (25). We were not able to establish whether gender influence survival and first-time MACE curves since only 23% of the recipients were female (Supplementary Figures S3, S4). Scandinavian results on long-term follow-up after heart transplantation (*n* = 2293) have documented no significant difference in survival when stratified by gender (*p* = 0.44) (19). However, this issue warrants further investigation.

Several previous studies have linked SEP to prognostic outcomes in heart-transplant recipients. A nationwide follow-up study in England, including 2,384 adult heart transplant-recipients (1995–2014) demonstrated that the most socioeconomically disadvantaged recipients had a 27% higher risk-adjusted 19-year overall mortality (HR 1.27, 95% CI 1.04–1.55). The United Kingdom multiple deprivation index was used to measure SEP (7). Similarly, a study (6) using the UNOS database in 36,736 adult (≥18 years) first-time heart-transplant recipients (1994–2014) found that college educated patients had an 18% reduced rate of deaths. Moreover, lowest SEP (index of seven SEP indicators) confers higher unadjusted risk of post-transplant hospitalization (HR 1.13), rejection (HR 1.28), infection (HR 1.10), and ischemic event (HR 1.26) (6). Another UNOS-based study including 5,125 primarily pediatric heart transplant recipients (2000–2011) reflected that risk adjusted survival was poorer in groups with a low SEP (HR 1.41, 95% CI 1.10–1.80) (26). Findings from a single-center Boston study among first-time heart transplant recipients (*n* = 520) conducted between 1996 and 2005 supported that low SEP (score of six variables) was associated with higher adjusted risk of graft loss (HR 1.5, 95% CI 1.0–2.4) (9). Findings from a previous UNOS analysis in left ventricular assist devices (LVAD) recipient’s ≥18 years (*n* = 3361) waiting for heart transplantation demonstrated that recipients with lower SEP (index of seven SEP indicators) had an early and sustained decreased adjusted post-transplant survival (lowest quartile: HR 0.57, 95% CI 0.39–0.82; highest quartile: HR 0.68, 95% CI 0.48–0.95 (8). Moreover, an analysis of the UNOS database including 33,893 adult heart-transplant recipients suggested an increased risk of mortality or re-transplantation (Adjusted *p* <0.001) associated with public health insurance status (Medicaid or Medicare versus private) (6). Research also based on the UNOS database studying a population (*n* = 20,676) of heart transplant recipients >17 years showed that Medicare and Medicaid insurances were associated with lower 10-year mortality risk (18%, 33%, respectively) than private insurance (10). In addition, multivariable analyses found that college-education decreased risk of mortality with 11% (10). In contrast to most previous studies using area-based social deprivation indexes or under-insurance status, we examined socioeconomic factors by individual and complete register-based single indicators of social vulnerability. Between 1 and 10 years post-surgery in particular, we observed a modest SEP gradient in the risk of a first-time MACE in heart transplant recipients. Remarkably, our results reflect that low educational attainment could be the most influential factor on both mortality and MACE, whereas personal income only influenced MACE. A recent single-center Danish study including 325 first-time heart transplant recipients (79% male and 69% between 41 and 60 years) described a lower median number of redeemed medical prescriptions during 15 years of follow-up in heart-transplant recipients within the lowest income group or if living alone (20). The association between income and prognosis could thus also be partly driven by an economic gradient in use of the prescribed medical treatment after heart transplantation. In line with the current understanding (6, 7, 9), it seems possible that even in a country with free access to multidisciplinary team management programs, educationally and economically disadvantaged heart transplant recipients could have an increased risk of non-adherence to immunosuppressive treatment, inadequate self-management skills, experience health disparities, and missed healthcare delivery; thus, graft failure and all-cause mortality are more likely in these recipients. However, our results indicate that the individual-level SEP impacts the middle follow-up period 1–10 years after transplantation. The most likely explanation for this is that socioeconomic disparities narrow over time after heart transplantation due to the role of the multidisciplinary team management identifying barriers to medical adherence and engaging patients to follow health recommendations. In accordance with the single-center Danish study, we believe that living alone may negatively influence on pharmalogical self-care. The lack of association between living alone and prognosis may be a result of the small sample size.

Our study also included information on comorbidities and chronic mental diseases 10 years prior to heart transplantation. Since the median number of comorbidities at baseline was one and less than 1% of the recipients had a psychiatric disorder, multimorbidity and psychiatric disorder were too rare to allow for further analyses of interactions. This may be explained by careful recipient selection based on pre- and post-transplant life expectancy, which reflects the recipient’s pre-operative psychosocial status and comorbidity burden (1, 2).

Remarkably, a prior UNOS study (27) in the United States (2001–2014) investigated the effect of non-working of heart transplant recipients (*n* = 23.228, >18 years) on survival. An adjusted analysis demonstrated a 5% and 10% decrease in 5- and 10-year mortality, respectively. Our study did not reveal any influence of occupational status. The most likely explanation is that our cohort included recipients >65 years (age at receiving state pension in Denmark) as well as the early retirement status of chronic end-stage heart failure recipients.

Although the Danish healthcare system appears to ensure easy access to multidisciplinary team management programs and fully funded immunosuppressive and medical treatment, our results support that in mainly educationally and economically disadvantaged recipients, the long-term prognosis of heart-transplant recipients is affected. This study contributes with knowledge to target long-term healthcare strategies for socially disadvantaged heart-transplant recipients across the world (16, 17). Our data suggests the need to focus on socioeconomic factors and their influence on both adherence and rehabilitation to support adequate self-management, self-efficacy, and health literacy after heart transplantation (5). The development of new mobile health devises (mHealth) in the field of transplantation has immense potential to facilitate healthcare service and implement more individualized education and management programs (28, 29). Further studies are needed to design and address delivery of more socially differentiated multidisciplinary team management programs for this patient group.

The setting in our study, including all heart transplant recipients in Denmark with long-term follow-up and individual accurate data linkage within a uniform healthcare system, reduced selection and recall bias. A critical limitation was that data in the DCDR (18) were not validated. Thus, the diagnosis of both underlying and contributory causes depends on the decision of the individual physician. We used a simple disease count algorithm to estimate the degree of multimorbidity. Thus, the relative severity of disease combinations was not assessed, and residual confounding could thus occur. Another limitation is the lack of precise temporality between baseline SEP and all-cause mortality or MACE, which does not allow inference from the identified observations. Notably, the combined MACE has not been validated. However, the component outcomes were validated in the general populations (18). Even though we adjusted our analysis for important confounding factors, residual confounding cannot be ruled out, since important clinical risk factors, blood sample measurements, and surgical procedure data were not available. Due to the small sample size, our reported associations should be supported in future large-scale observational studies.

We found that in first-time heart transplant recipients, the half-time survival was 15.6 years during a 20-year period. Low levels of education and income were associated with a poorer prognosis after surgery despite selection during the assessment process leading to heart transplantation.

## Data Availability

Study data, statistical plan, and log-files can be made available through proposal to the Project Database (ID: 707738) at Statistics Denmark. https://www.dst.dk/en/TilSalg/Forskningsservice.

## Abbreviations

ATC, anatomical therapeutic chemical; CRS, civil registration system; DCDR, Danish causes of death registry; DNPR, Danish national patient registry; MACE, major adverse cardiovascular event; NPR, Danish national prescription registry; PCRR, psychiatric central research registry; SEP, socioeconomic position; STD, Scandiatransplant database.
