# Supplementary material for: Individual-Level Socioeconomic Position and Long-Term Prognosis in Danish Heart-Transplant Recipients
Source: Transpl Int. 2023 Mar 22;36:10976. doi: 10.3389/ti.2023.10976 (PMC10073462; doi:10.3389/ti.2023.10976)
Supplement: Supplementary file 1 [file DataSheet1.PDF]

SUPPORTING INFORMATION

Individual-level Socioeconomic Position and Long-term Prognosis in Danish Heart-transplant Recipients

Contents

TABLE S1 International Classification of Disease of comorbidities

TABLE S2 Anatomical Therapeutic Chemical Classification Codes of cardiovascular medical treatment

TABLE S3 International Classification of Diseases Codes of algorithm to measure multimorbidity

TABLE S4 Classification of individual-level socioeconomic position

TABLE S5 International Classification of Disease regarding cause of mortality

TABLE S6 International Classification of Disease and Nordic Medio-statistical Committee's Classification of Surgical Procedures

TABLE S7 Standardized biopsy controls after heart transplantation (Index date)

TABLE S8 Crude and adjusted HRs for all-cause mortality

TABLE S9 Crude and adjusted HRs first-time MACE

FIGURE S1 Long-term survival by time era

FIGURE S2 Long-term conditional 1-year post-survival by time era

FIGURE S3 Long-term survival by gender

FIGURE S4 Long-term first-time MACE by gender

TABLE S1 International Classification of Disease of comorbidities (1-4)

**Comorbidities**	ICD-8*	ICD-10*
**Cardiovascular**		
Myocardial infarction	410	I21
Angina pectoris		
Unstable	411	I200
Stable	413	I20 (without I200), I251, I259
Heart Failure	42709, 42710, 42711, 42719, 42899,78249	I500, I501, I502, I503, I508, I509, I110, I130, I132, I420, I426, I427, I428, I429
Heart valve disease		
Mitral valve-insufficiency and stenosis	394	I05, I34, I390, I511A
Aorticvalve-insufficiency and stenosis	395	I06, I35, I391
Cardiac arrhythmia		
Atrial fibrillation or flutter	42793, 42794	I48
Bradycardia (sinus node dysfunction and AV-block)	42720, 42721, 42722, 42723	I440, I441, I442, I443, use 145 (*I455A, I455B, I455C, I455G)*<br>I470, I472, I490
Ventricular tachycardia/fibrillation	42797, 42791	
Congenital heart disease	746.00–747.49, 759.00, 759.01, 759.09	Q200-Q269, Q893
Cardiomyopathy	425	I42-I43 (excluding I42.6)
Cardiac inflammation		
Endocarditis	421	I33, I38, I398
Myocarditis	422	I40, I41, I090, I514
Pericarditis	39109, 393, 420, 423	I30-132
Aortic disease		
Aortadissection	44109	I710
Aneurisme/dilatation	44110, 44111, 44119, 44120, 44121, 44129, 44199	I711-I716, I718-I719
Peripheral arterial disease	44389-44399	I739A
Cerebrovascular disease	430-438	I60-I69, G45, G46
Cardiogenic shock and pulmonary edema	427.10, 427.11	J81, I501B, R570
**Other**	**ICD-8**	**ICD-10**
Diabetes with end organ damage type1 type2	249.00, 249.06, 249.07, 249.09, 250.00, 250.06, 250.07, 250.09	E10-E14. O24 (except O24.4), G63.2, H36.0, N08.3
Hypertension	400-404	I10-I15
Chronic obstructive pulmonary disease	490-493; 515-518	J40–J47; J60–J67; J68.4; J70.1; J70.3; J84.1; J92.0; J96.1; J98.2; J98.3
Obesity	277	E65-E68

Psychiatric disorder		
Schizophrenia	295.x9, 296.89,	F20 –F29
And related disorders/psychotic disorders	297.x9, 298.29-298.99, 299.04, 299.05, 299.09, 301.83	
And related disorders/psychotic disorders		
Affective disorders incl. depression	296.x9 (excluding 296.89), 298.09, 298.19, 300.49, 301.19	F30-39
Dementia		
Alzheimer's disease	290.10	F00 (includes F00.0x, F00.1x, F00.2x, and F00.9x);<br>G30 (includes G30, G30.0, 30.1, 30.8, 30.9)
Vascular dementia	293.09, 293.19	F01 (includes F01.0x, F01.1x, F01.2x, F01.3x, F01.8x, & F01.9x)
Other dementia	094.19 and 292.09; 290.09, 290.11, 290.18, 290.19, 292.09	F02; F03; F05.1; F1x.73 (F10.73 through F19.73);<br>G23.1; G31.0A*, G31.0B*, G31.1, G31.8B, G31.8E
Other exclusions: Mild cognitive impairment (MCI) and amnestic syndromes. Applies when identifying cases and controls free from dementia. Not used to identify incident dementia	291.19	F04, F04.9, F05.1, F06.7 and F06.7x;<br>F1x.6 (F10.6, F18.6, F19.6)
Neurotic, stress-related and somatoform disorder	300.x9 (excluding 300.49), 305.x9, 305.68, 307.99	F40-F48
Eating disorder	305.60, 306.50, 306.58, 306.59	F50

Abbreviations: ICD, International Classification of Disease.
* Due to our time perspective before 1995, we added ICD-8 codes. We obtained information on diagnosis (both primary and secondary) from inpatient and outpatient hospital diagnoses (International Classification of Disease: ICD-8 & ICD-10) recorded in the Danish National Patient Registry (DNPR) 10 years prior to the index date.

1. Schmidt M, Schmidt SA, Sandegaard JL, Ehrenstein V, Pedersen L, Sorensen HT. The Danish National Patient Registry: a review of content, data quality, and research potential. Clinical epidemiology 2015;7:449-90.
2. Sundbøll J, Adelborg K, Munch T et al. Positive predictive value of cardiovascular diagnoses in the Danish National Patient Registry: a validation study. BMJ open 2016;6:e012832.
3. Adelborg K, Sundbøll J, Munch T et al. Positive predictive value of cardiac examination, procedure and surgery codes in the Danish National Patient Registry: a population-based validation study. BMJ open 2016;6:e012817.
4. Mors O, Perto GP, Mortensen PB. The Danish Psychiatric Central Research Register. Scandinavian journal of public health 2011;39:54-7.

TABLE S2 Anatomical Therapeutic Chemical Classification Codes of cardiovascular medical treatment (1)

Cardiovascular medical treatment	ATC Classification
Cardiac therapy	C01
Anti-hypertensive	CO2
Diuretics	CO3
Peripheral vasodilators	CO4
Vasoprotective	CO5
Beta-blockers	CO7
Calcium antagonists	CO8
Renin-angiotensin system inhibitions	CO9
Lipid-modifying	CO10
Anticoagulants	B01A
Aspirin	B01AC06

Abbreviations: ACT, Anatomical Therapeutic Chemical Classification.

1. Pottegard A, Schmidt SAJ, Wallach-Kildemoes H, Sorensen HT, Hallas J, Schmidt M. Data Resource Profile: The Danish National Prescription Registry. International journal of epidemiology 2017;46:798-798f.

TABLE S3 International Classification of Diseases Codes of algorithm to measure multimorbidity (1-4)

Included CDGs (grey) and included diseases (white)	ICD-8	ICD-10
**Cardiovascular disease (5,6)**		
Myocardial infarction	410	I21
Angina Pectoris		
Unstable	411	I200
Stable	413	I20 (without I200), I251, I259
Heart Failure	42709, 42710, 42711, 42719, 42899,78249	I500, I501, I502, I503, I508, I509, I110, I130, I132, I420, I426, I427, I428, I429
Heart Vale disease		
Mitralvalve-insufficiency and stenosis	394	I05, I34, I390, I511A
Aorticvalve-insufficiency and stenosis	395	I06, I35, I391
Cardiac arrhythmia		
Atrial fibrillation or flutter	42793, 42794	I48
Bradycardia (sinus node dysfunction and AV-block)	42720, 42721, 42722, 42723	I440, I441, I442, I443, use 145 (*I455A, I455B, I455C, I455G)*
Ventricular tachycardia/fibrillation	42797, 42791	I470, I472, I490
Cardiac inflammation		
Endocarditis	421	I33, I38, I398
Myocarditis	422	I40, I41, I090, I514
Pericarditis	39109, 393, 420, 423	I30-132
Aortic disease		
Aortadissection	44109	I710
Aneurisme/dilatation	44110, 44111, 44119, 44120, 44121, 44129, 44199	I711-I716, I718-I719
Peripheral arterial disease	44389-44399	I739A
Cerebrovascular disease	430-438	I60-I69, G45, G46
Cardiogenic shock and pulmonary edema	427.10, 427.11	J81, I501B, R570
Congenital heart disease	746.00–747.49, 759.00, 759.01, 759.09	Q200-Q269, Q893
Cardiomyopathy	425	I42-I43 (excluding I42.6)
**Hypertension**		
Hypertension	400-404	I10-I15

**Diabetes**		
Diabetes with end organ damage type1 type2	249.00, 249.06, 249.07, 249.09, 250.00, 250.06, 250.07, 250.09	E10-E14. O24 (except O24.4), G63.2, H36.0, N08.3
**Chronic obstructive pulmonary disease (5,6)**		
COPD	490-493; 515-518	J40–J47; J60–J67; J68.4; J70.1; J70.3; J84.1; J92.0; J96.1; J98.2; J98.3
**Cancer (5,6)**		
Cancer	140–209	C00-C97
**Chronic neurological disorders (6)**		
Epilepsy	345	G40 (ex. G40.4), G41
Parkinson's disease	342	G20-G22
Multiple sclerosis	340	G35
**Chronic arthritis (6)**		
Rheumatoid arthritis/connective tissue disease	696.09, 712, 715	L40.5, M05-M07
**Inflammatory bowel disease/Chronic bowel disease (6)**		
Colitis ulcerosa and Mb. Crohn	563	K50-K51
**Chronic liver disease (6)**		
Chronic viral hepatitis,	571-573	B18
Chronic liver disease		K70-K76
**Chronic kidney disease (6)**		
Chronic pyelonephritis/Interstitial nephritis	590.09, 593.20	N11, N14, N15, N16
Other and unknown chronic renal disease (none of those above, but):	792, 584	N18-N19, N26, N27, N07, N08 (without N08.3)
**Psychiatric disorder (5,6)**		
Schizophrenia	295.x9, 296.89,	F20 –F29
And related disorders/psychotic disorders	297.x9, 298.29-298.99, 299.04, 299.05, 299.09, 301.83	
Affective disorders incl. depression	296.x9 (excluding 296.89), 298.09, 298.19, 300.49, 301.19	F30-39
Dementia		
Alzheimer's disease	290.10	F00 (includes F00.0x, F00.1x, F00.2x, and F00.9x); G30 (includes G30, G30.0, 30.1, 30.8, 30.9)

Vascular dementia	293.09, 293.19	F01 (includes F01.0x, F01.1x, F01.2x, F01.3x, F01.8x, & F01.9x)
Other dementia	094.19 and 292.09; 290.09, 290.11, 290.18, 290.19, 292.09	F02; F03; F05.1; F1x.73 (F10.73 through F19.73); G23.1; G31.0A*, G31.0B*, G31.1, G31.8B, G31.8E
Other exclusions: Mild cognitive impairment (MCI) and amnestic syndromes. Applies when identifying cases and controls free from dementia. Not used to identify incident dementia	291.19	F04, F04.9, F05.1, F06.7 and F06.7x; F1x.6 (F10.6, F18.6, F19.6)
Neurotic, stress-related and somatoform disorder	300.x9 (excluding 300.49), 305.x9, 305.68, 307.99	F40-F48
Eating disorder	305.60, 306.50, 306.58, 306.59	F50

Abbreviations: CDG, Comprehensive groups of Chronic Diseases; ICD, International Classification of Disease.

1. Schmidt M, Schmidt SA, Sandegaard JL, Ehrenstein V, Pedersen L, Sorensen HT. The Danish National Patient Registry: a review of content, data quality, and research potential. Clinical epidemiology 2015;7:449-90.
2. Sundbøll J, Adelborg K, Munch T et al. Positive predictive value of cardiovascular diagnoses in the Danish National Patient Registry: a validation study. BMJ open 2016;6:e012832.
3. Adelborg K, Sundbøll J, Munch T et al. Positive predictive value of cardiac examination, procedure and surgery codes in the Danish National Patient Registry: a population-based validation study. BMJ open 2016;6:e012817.
4. Mors O, Perto GP, Mortensen PB. The Danish Psychiatric Central Research Register. Scandinavian journal of public health 2011;39:54-7.
5. Diederichs C, Berger K, Bartels DB. The measurement of multiple chronic diseases--a systematic review on existing multimorbidity indices. The journals of gerontology Series A, Biological sciences and medical sciences 2011;66:301-11.
6. Barnett K, Mercer SW, Norbury M, Watt G, Wyke S, Guthrie B. Epidemiology of multimorbidity and implications for health care, research, and medical education: a cross-sectional study. Lancet (London, England) 2012;380:37-43.

TABLE S4 Classification of individual-level socioeconomic position (1-5)

**Individual-level socioeconomic position**		**Dichotomized**
Cohabitation status	Alone (single, divorced, widow, etc.)<br>Cohabitating (married or in registered partnership)	**Alone**<br>**Cohabitating**
Educational degree	Low-degree (no formal education; primary and lower secondary education)<br>Medium-degree (upper secondary education and academy profession)<br>High-degree (bachelor and above)<br>Not completed education (patients under age of 16 years)<br>Missing	**Low-degree** (low degree)<br>**Medium-high-degree** (medium + high degree)<br><br>(*Patients <16 years not included)*<br>*(Missing not included)*
Personal income	Based on the annually reported (since 1995) nationwide personal (pre-tax total) income statistics, we selected the 25th, 50th and 75th percentiles and then used the 25th percentile as a cut-off value for a binary income categorization: lowest (≤ 25th percentile) and medium-high (> 25th percentile).<br><br>Data on personal (pre-tax total) income were obtained from the Income Statistics Register (5)<br>The annual personal (pre-tax-total) income 25th, 50th and 75th percentiles in the National Danish Population was available from 1995, and thus income was estimated by linear regression from 1993-1995. They are based on the numbers downloaded from the STATBAK.DK at January 13, 2021.<br><br>https://www.statbank.dk/statbank5a/SelectTable/Omrade0.asp?SubjectCode=04&ShowNews=OFF&PLanguage=1	**Low** (≤25th percentile)<br>**Medium-high** (>25th percentile)
Occupational status	Working<br>Non-working (no employment or early retirement)<br>Out-of-workforce (state pension, under education)<br>Missing	**Unemployed** (non-working)<br>**Employed** (working, out-of-workforce)<br><br>*(Missing not included)*

1. Galobardes B, Shaw M, Lawlor DA, Lynch JW, Davey Smith G. Indicators of socioeconomic position (part 1). Journal of epidemiology and community health 2006;60:7-12.
2. Schmidt M, Pedersen L, Sorensen HT. The Danish Civil Registration System as a tool in epidemiology. European journal of epidemiology 2014;29:541-9.
3. Petersson F, Baadsgaard M, Thygesen LC. Danish registers on personal labour market affiliation. Scandinavian journal of public health 2011;39:95-8.
4. Jensen VM, Rasmussen AW. Danish Education Registers. Scandinavian journal of public health 2011;39:91-4.
5. Baadsgaard M, Quitzau J. Danish registers on personal income and transfer payments. Scandinavian journal of public health 2011;39:103-5.

TABLE S5 International Classification of Disease regarding cause of mortality (1)

Underlying, intermediate, and contributory causes (ICD-10 codes)		Classification
T85	Complications of other internal prosthetic devices, implants and grafts	Complications of heart transplantation
T86	Complications of transplanted organs and tissue	Complications of heart transplantation
T82	Complications of cardiac and vascular prosthetic devices, implants and grafts	Complications of heart transplantation
A419	Multiple organ failure (sepsis)	Multiple organ failure
I132	Hypertensive heart and renal disease with both (congestive) heart failure and renal failure	Multiple organ failure
I12	Hypertensive renal disease with renal failure	Multiple organ failure
K726	Hepatic failure, not elsewhere classified	Multiple organ failure
R092A	Respiratory arrest	Multiple organ failure
I971	Postprocedural disorders of circulatory system, not elsewhere classified	Multiple organ failure
O742A	Complications of anaesthesia during labour and delivery	Multiple organ failure
J85	Gangrene and necrosis of lung	Multiple organ failure
R57	Cardiogenic shock	Sudden death
I46	Cardiac arrest	Sudden death
R98	Unattended death	Sudden death
I24	Other acute ischaemic heart diseases	Sudden death
I26	Pulmonary embolism with mention of acute cor pulmonale	Sudden death
I64	Stroke, not specified as haemorrhage or infarction	Sudden death

X60-X64	Intentional self-harm	Sudden death
I20	Angina pectoris	Cardiovascular disease
I21	Acute myocardial infarction	Cardiovascular disease
I22	Subsequent myocardial infarction	Cardiovascular disease
I25	Chronic ischemic heart disease	Cardiovascular disease
I49	Ventricular fibrillation and flutter	Cardiovascular disease
I71	Dissection of aorta	Cardiovascular disease
I7O	Atherosclerosis	Cardiovascular disease
I72	Other aneurysm and dissection	Cardiovascular disease
I73	Other peripheral vascular diseases	Cardiovascular disease
I74	Arterial embolism and thrombosis	Cardiovascular disease
I31	Other diseases of pericardium	Cardiovascular disease
I10	Essential (primary) hypertension	Cardiovascular disease
I11	Hypertensive heart disease	Cardiovascular disease
I35	Nonrheumatic aortic valve disorders	Cardiovascular disease
I50	Heart failure	Heart failure
I42	Cardiomyopathy	Heart failure
I13	Hypertensive heart and renal disease with (congestive) heart failure	Heart failure
J81	Pulmonary oedema	Heart failure
I60	Subarachnoid haemorrhage	Cerebrovascular disease

I61	Nontraumatic intracerebral hemorrhage	Cerebrovascular disease
I62	Other nontraumatic intracranial haemorrhage	Cerebrovascular disease
I63	Cerebral infarction	Cerebrovascular disease
G93	Other disorders of brain	Cerebrovascular disease
A41	Sepsis due to Staphylococcus aureus	Infection
J15	Bacterial pneumonia, not elsewhere classified	Infection
J12	Viral pneumonia, not elsewhere classified	Infection
J15	Bacterial pneumonia, not elsewhere classified	Infection
J18	Pneumonia, organism unspecified	Infection
J84	Other interstitial pulmonary diseases	Infection
J85	Abscess of lung and mediastinum	Infection
A04	Other bacterial intestinal infections	Infection
A49	Bacterial infection of unspecified site	Infection
A46	Erysipelas	Infection
B00	Herpesviral [herpes simplex] infections	Infection
B25	Cytomegaloviral disease (pneumoni)	Infection
B44	Aspergillosis	Infection
B49	Unspecified mycosis	Infection
B59	Pneumocystosis	Infection
B99	Other and unspecified infectious diseases	Infection

J69	Pneumonitis due to food and vomit	Infection
N39	Urinary tract infection, site not specified	Infection
I38	Endocarditis, valve unspecified	Infection
I40	Acute myocarditis	Infection
G03	Meningitis due to other and unspecified causes	Infection
G04	Encephalitis, myelitis and encephalomyelitis	Infection
J15	Bacterial pneumonia, not elsewhere classified	Infection
J18	Pneumonia, unspecified organism	Infection
I26	Pulmonary embolism with mention of acute cor pulmonale	Pulmonary disease
J44	Other chronic obstructive pulmonary disease	Pulmonary disease
J80	Adult respiratory distress syndrome	Pulmonary disease
J90	Pleural effusion, not elsewhere classified	Pulmonary disease
J93	Pneumothorax	Pulmonary disease
J96	Acute respiratory failure	Pulmonary disease
C00-C97	Cancer	Malignancy
N05	Unspecified nephritic syndrome	Kidney disease
N14	Drug- and heavy-metal-induced tubulo-interstitial and tubular conditions	Kidney disease
N17	Acute renal failure with tubular necrosis	Kidney disease
N18	Chronic kidney disease (CKD)	Kidney disease
N19	Unspecified kidney failure	Kidney disease

N28	Other disorders of kidney and ureter, not elsewhere classified	Kidney disease
N39	Other disorders of urinary system	Kidney disease
Z49	Care involving dialysis	Kidney disease
E10-E14	Diabetes mellitus	Diabetes
Z94	Liver transplant status	Other specified
Z95	Presence of cardiac and vascular implants and grafts	Other specified
R09	Other symptoms and signs involving the circulatory and respiratory system	Other specified
K55	Vascular disorders of intestine	Other specified
D64	Other anaemias	Other specified
E83	Disorders of mineral metabolism	Other specified
K22	Other diseases of oesophagus	Other specified
K25	Gastric ulcer	Other specified
K52	Other noninfective gastroenteritis and colitis	Other specified
K55	Vascular disorders of intestine	Other specified
K56	Other and unspecified intestinal obstruction	Other specified
R10	Acute abdomen	Other specified
D89	Other disorders involving the immune mechanism, not elsewhere classified	Other specified
R99	Defined and unknown cause of mortality	Not specified
A469	Erysipelas (Certain infectious and parasitic diseases)	Not specified
I45	Right fascicular block	Not specified

I47	Supraventricular tachycardia	Not specified
I49	Other cardiac arrhythmias	Not specified
I51	Cardiac septal defect, acquired	Not specified
R64	Cachexia	Not specified
D86	Sarcoidosis of lung	Not specified
Q20	Congenital malformations of cardiac chambers and connections	Not specified
Q23	Congenital malformations of aortic and mitral valves	Not specified
Q24	Dextrocardia	Not specified
Q25	Congenital malformations of great arteries	Not specified
R00-R99	Symptoms and signs involving the circulatory and respiratory systems	Not specified
T43	Tricyclic and tetracyclic antidepressants	Not specified
T58	Toxic effect of carbon monoxide	Not specified
T81	Complications of procedures, not elsewhere classified	Not specified
T82	Toxic effect of carbon monoxide	Not specified
T87	Complications peculiar to reattachment and amputation	Not specified
T88	Other complications of surgical and medical care, not elsewhere classified	Not specified
F03	Manic episode	Not specified
F15	Mental and behavioural disorders due to use of other stimulants, including caffeine	Not specified
F32	Depressive episode	Not specified
G12	Spinal muscular atrophy and related syndromes	Not specified

G04	Epilepsy	Not specified
S06	Intracranial injury	Not specified
S92	Other and unspecified injuries of thorax	Not specified
S08	Traumatic amputation of unspecified part of head	Not specified
W00-W19	Falls	Not specified
W23	Caught, crushed, jammed or pinched in or between objects	Not specified
E85	Non-neuropathic heredofamilial amyloidosis	Not specified
E87	Other disorders of fluid, electrolyte and acid-base balance	Not specified
K70	Alcoholic liver disease	Not specified
K72	Fracture of femur	Not specified
K80	Cholelithiasis	Not specified
K91	Postprocedural disorders of digestive system, not elsewhere classified	Not specified
K92	Other diseases of digestive system	Not specified
N99	Postprocedural disorders of genitourinary system, not elsewhere classified	Not specified
X59	Exposure to unspecified factor	Not specified
X67	Intentional self-poisoning by and exposure to other gases and vapours	Not specified
X74	Intentional self-harm by other and unspecified firearm discharge	Not specified
Y11	Poisoning by and exposure to antiepileptic, sedative-hypnotic, antiparkinsonism and psychotropic drugs, not elsewhere classified, undetermined intent	Not specified
Y88	Sequelae with surgical and medical care as external cause	Not specified
Q22	Venous complications and haemorrhoids in pregnancy	Not relevant

Abbreviations: ICD, International Classification of Disease.

1. Helweg-Larsen K. The Danish Register of Causes of Death. Scandinavian journal of public health 2011;39:26-9.

TABLE S6 International Classification of Disease and Nordic Medio-statistical Committee's Classification of Surgical Procedures (1-3)

Events	ICD-8, ICD-10, NOMESCO
Myocardial infarction	I21
Peripheral arterial disease	I739A
Cardia arrest	I46
Stroke	I63-I64, G459
Cardiac inflammation	
Endocarditis	I33, I38, I398
Myocarditis	I40, I41, I090, I514
Pericarditis	I30-132
Readmission due to heart failure	I500, I501, I502, I503, I508, I509, I110, I130, I132, I420, I426, I427, I428, I429
Graft failure (heart-transplant failure and rejection)	T862
Percutaneous Coronary Intervention (unspecified with/without stent)	Before 1996: 30350, 30354, 30240 (Maybe not necessary to use)<br>After 1996: procedure code: KFNG, KFN
Radiofrequency ablation for atrial fibrillation	BFFB
Cardiac pacemaker	BFCA0, BFCA6, KFPE00, KFPE10, KFPE20, KFPE96, KFPF00, KFPF10, KFPF20, KFPF96<br>BFCB0, BFCB6, KFPG
Valve surgery	Before 1996: 30300, 30310, 30320, 30330, 30340, 30350, 30360, 30600, 30620, 30640, 30660, 30700, 30701, 30709, 30719, 30720, 30729, 30740, 30780, 30799, 30800, 30810, 30910, 30920, 30925, 30939, 30959, 30990, 31100, 31101, 31119, 31129, 31130, 31180, 31199, 31200, 31210, 31220, 31229, 31230, 31249, 31259, 31268, 31269, 31280, 31299, 31310<br>After 1996: KFK, KFM
Heart transplantation	KFQA

Abbreviations: ICD, International Classification of Disease; NOMESCO, Nordic Medio-statistical Committee's Classification of Surgical Procedures since 1996.

1. Schmidt M, Schmidt SA, Sandegaard JL, Ehrenstein V, Pedersen L, Sorensen HT. The Danish National Patient Registry: a review of content, data quality, and research potential. Clinical epidemiology 2015;7:449-90.
2. Sundbøll J, Adelborg K, Munch T et al. Positive predictive value of cardiovascular diagnoses in the Danish National Patient Registry: a validation study. BMJ open 2016;6:e012832.
3. Adelborg K, Sundbøll J, Munch T et al. Positive predictive value of cardiac examination, procedure and surgery codes in the Danish National Patient Registry: a population-based validation study. BMJ open 2016;6:e012817.

TABLE S7 Standardized biopsy controls after heart transplantation (Index date)

**Biopsy controls**	0 - 6 week after index date	Weekly
	7 - 12 week after index date	Every second week
	3 – 6 months after index date	Every months yes
	6 - 12 months after index date	Aarhus: Every second months<br>Copenhagen: 1 + 12 months
	2 years after index date	

TABLE S8 Crude and adjusted HRs for all-cause mortality

	Mortality, N	Person-years	Crude HR (95% CI)	Adjusted HR (95% CI)
**Low education versus medium-high education (ref.)**				
0-1 year	18	158.6	0.80 (0.46-1.36)	0.84 (0.49-1.45)
1-10 years	38	1012.6	1.34 (0.89-2.02)	1.34 (0.88-2.02)
10-20 years	29	329.4	1.90 (1.18-3.05)	1.95 (1.19-3.19)
0-20 years	85	1500.6	1.29 (0.99-1.69)	1.34 (1.02-1.76)
**Living alone versus cohabitation (ref.)**				
0-1 year	21	207.4	0.70 (0.42-1.18)	0.73 (0.42-1.27)
1-10 years	39	1183.4	1.14 (0.76-1.70)	1.33 (0.86-2.04)
10-20 years	21	378.2	0.93 (0.56-1.54)	1.37 (0.79-2.39)
1-20 years	81	1769	0.94 (0.72-1.22)	1.13 (0.85-1.51)
**Low income versus medium-high income (ref.)**				
0-1 year	8	73.2	0.88 (0.42-1.83)	0.94 (0.44-1.99)
1-10 years	14	427	1.07 (0.61-1.88)	1.18 (0.66-2.11)
10-20 years	10	158.6	1.04 (0.53-2.02)	1.08 (0.55-2.11)
1-20 years	32	658.8	1.00 (0.69-1.45)	0.90(0.68-1.18)
**Unemployed versus employed (ref.)**				
0-1 year	26	207.4	0.93 (0.57-1.52)	0.89 (0.54-1.47)

1-10 years	37	1183.4	1.05 (0.70-1.58)	1.04 (0.68-1.58)
10-20 years	19	378.2	0.81 (0.48-1.37)	0.68 (0.39-1.19)
1-20 years	82	1769	0.94 (0.72-1.23)	0.90 (0.68-1.18)

Abbreviations: HR, Hazard Ratio; HRs, Hazard Ratios; CI, Confidence Intervals.

Cox proportional hazard models for adjusted HRs for all-cause mortality within follow-up intervals: 0-1 year, >1-10 years, and >10-20 years after heart transplantation in Denmark (1994-2018) according to dichotomized socioeconomic factors. In multivariate analysis, the HRs are adjusted for age, gender, donor age, donor mismatch, hypertension, and diabetes.

TABLE S9 Crude and adjusted HRs for first-time MACE

	MACE, N	Person-years	Crude<br>HR (95% CI)	Adjusted<br>HR (95% CI)
**Low education versus medium-high education (ref.)**				
1-10 years	48	451.4	1.65 (1.14-1.19)	1.66 (1.14-2.43)
10-20 years	8	97.6	1.74 (0.76-3.99)	2.58 (1.05-6.64)
1-20 years	56	549	1.67 (1.19-2.35)	1.71 (1.20-2.42)
**Living alone versus cohabitation (ref.)**				
1-10 years	49	524.6	1.39 (0.96-.2.01)	1.46 (0.98-2.17)
10-20 years	6	146.4	0.66 (0.27-1.64)	0.95 (0.34-2.64)
1-20 years	55	658.8	1.23 (0.88-1.73)	1.35 (0.94-1.95)
**Low income versus medium-high income (ref.)**				
1-10 years	45	134.2	1.48 (0.86-2.54)	1.81 (1.02-3.22)
10-20 years	2	48.8	0.77 (0.18-3.24)	0.62 (0.10-3.69)
1-20 years	47	183	1.33 (0.80-2.21)	1.67 (0.97-2.87)
**Unemployed versus employed (ref.)**				
1-10 years	45	536.8	1.14 (0.78-1.66)	1.28 (0.86-1.91)
10-20 years	5	134.2	0.61 (0.23-1.59)	0.64 (0.21-1.91)
1-20 years	50	671	1.04 (0.73-1.46)	1.18 (0.81-1.71)

Abbreviations: HR, Hazard Ratio; CI, Confidence Intervals; HRs, Hazard Ratios; MACE, Major Cardiovascular Event (composite of readmission due to heart failure, graft failure, percutaneous coronary intervention (overall), and all-cause mortality).

Cox proportional hazard models for adjusted HRs for first-time MACE within follow-up intervals: >1-10 years, and >10-20 years after heart transplantation in Denmark (1994-2018) according to dichotomized socioeconomic factors. In multivariate analysis, the HRs are adjusted for age, gender, donor age, donor mismatch, hypertension, and diabetes.

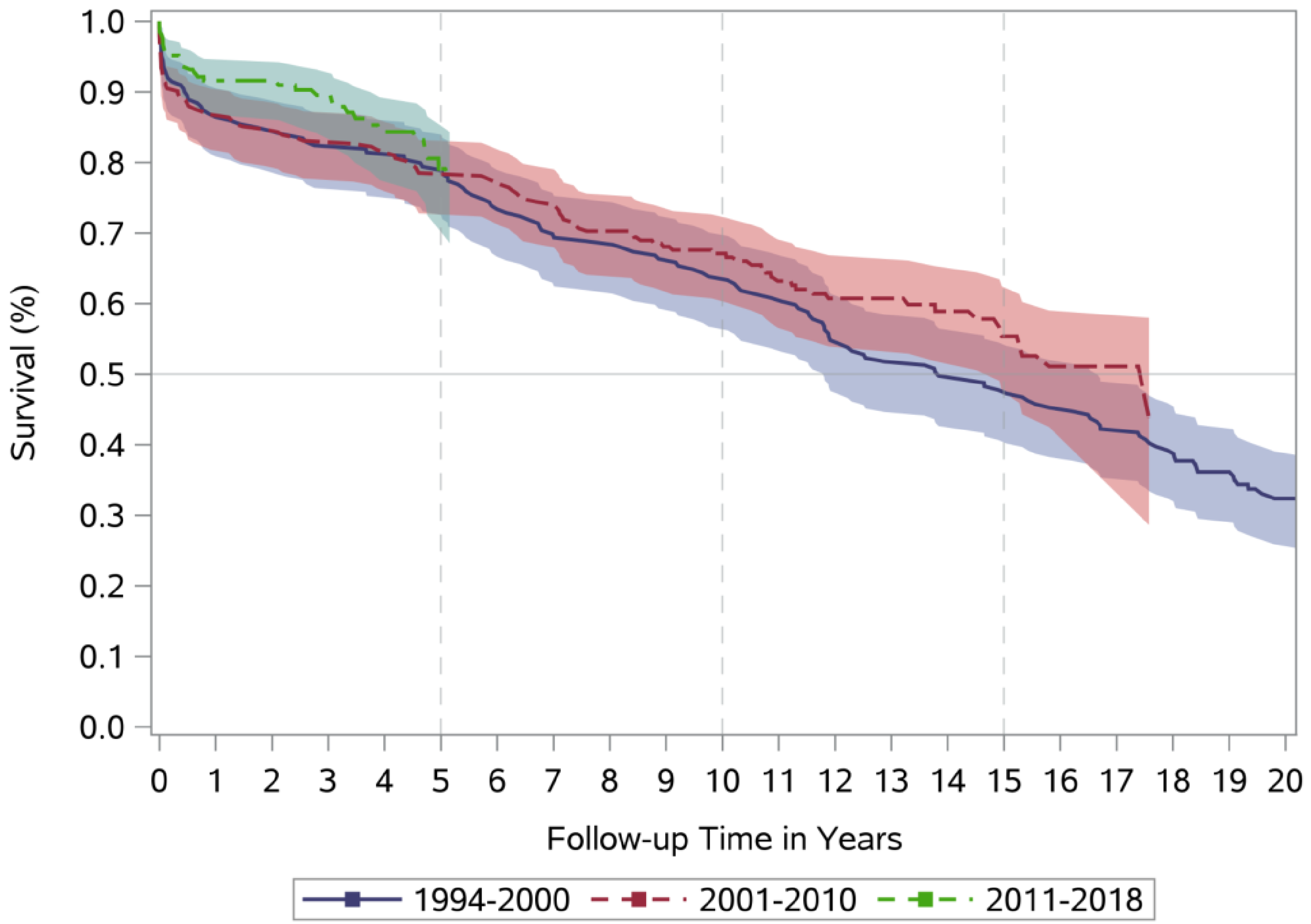

FIGURE S1 Long-term survival by time era

All-cause mortality after surgery date (index date) stratified according to time era (1994-2000, 2001-2010, 2011-2018)

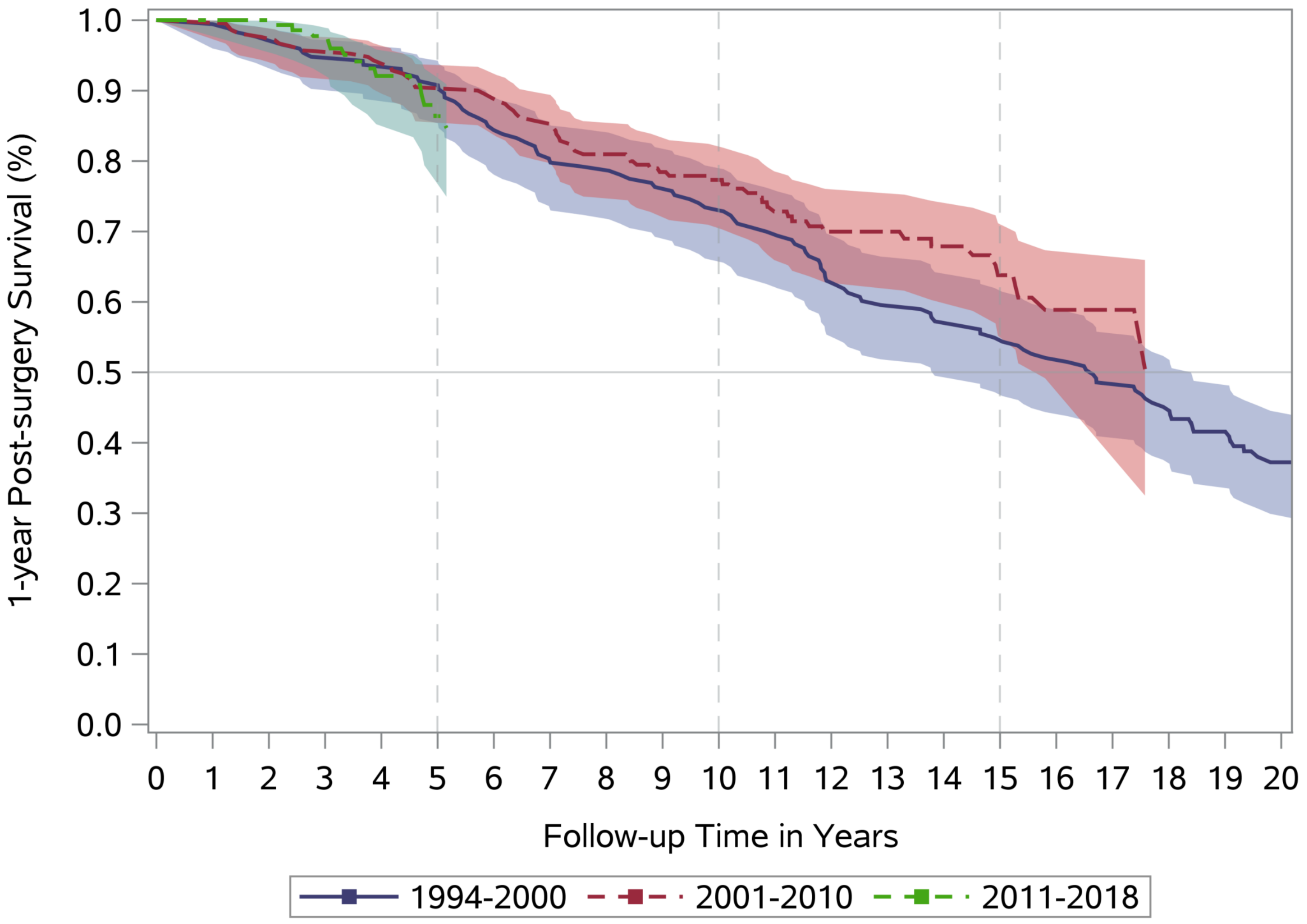

FIGURE S2 Long-term conditional 1-year post-survival by time era

1-year Post-surgery Survival, Conditional all-cause mortality in recipients who survived first year after the index date stratified according to time era (1994-2000, 2001-2010, 2011-2018).

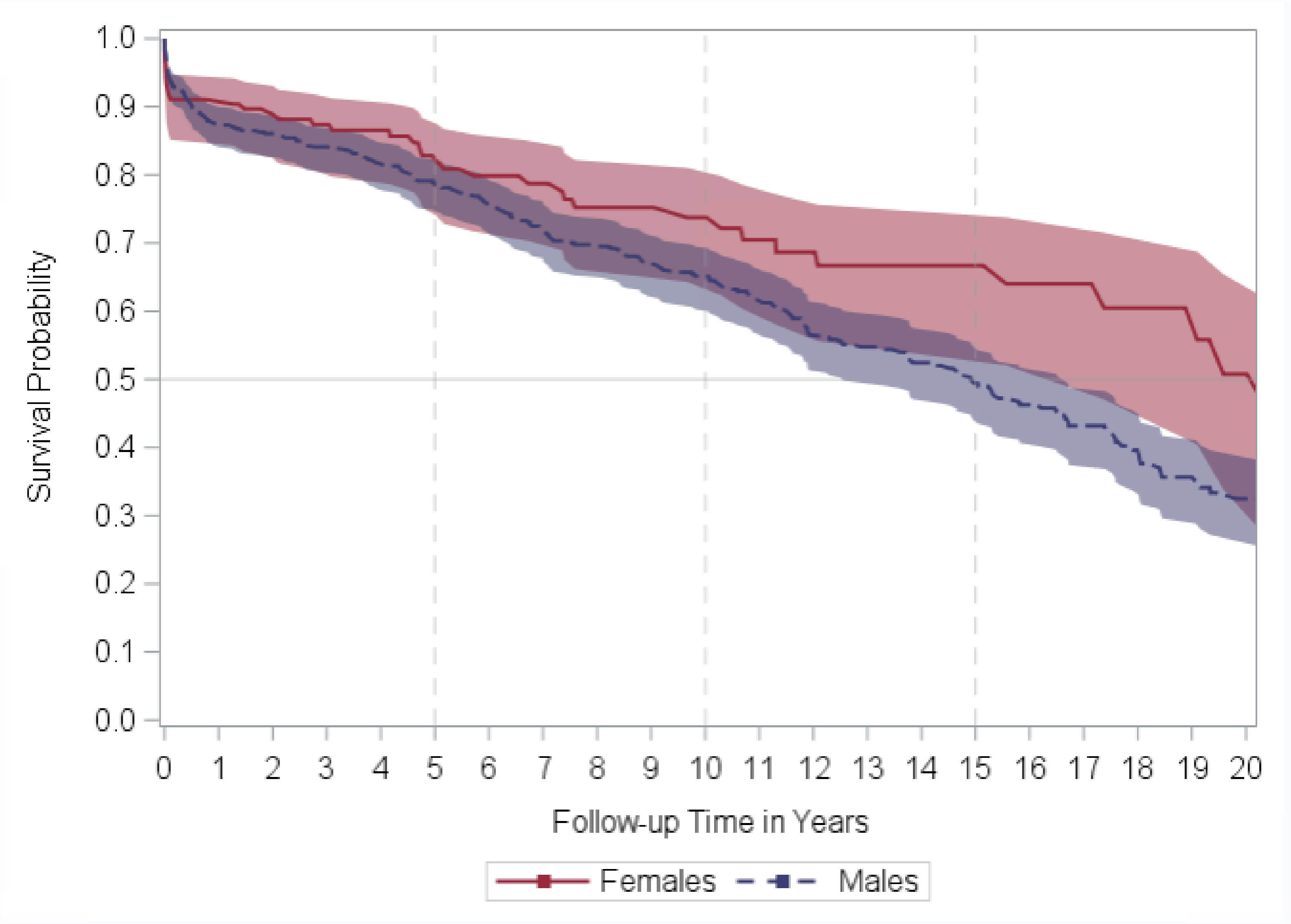

FIGURE S3 Long-term survival by gender

All-cause mortality after surgery date (index date) stratified by female (red) and male (blue)

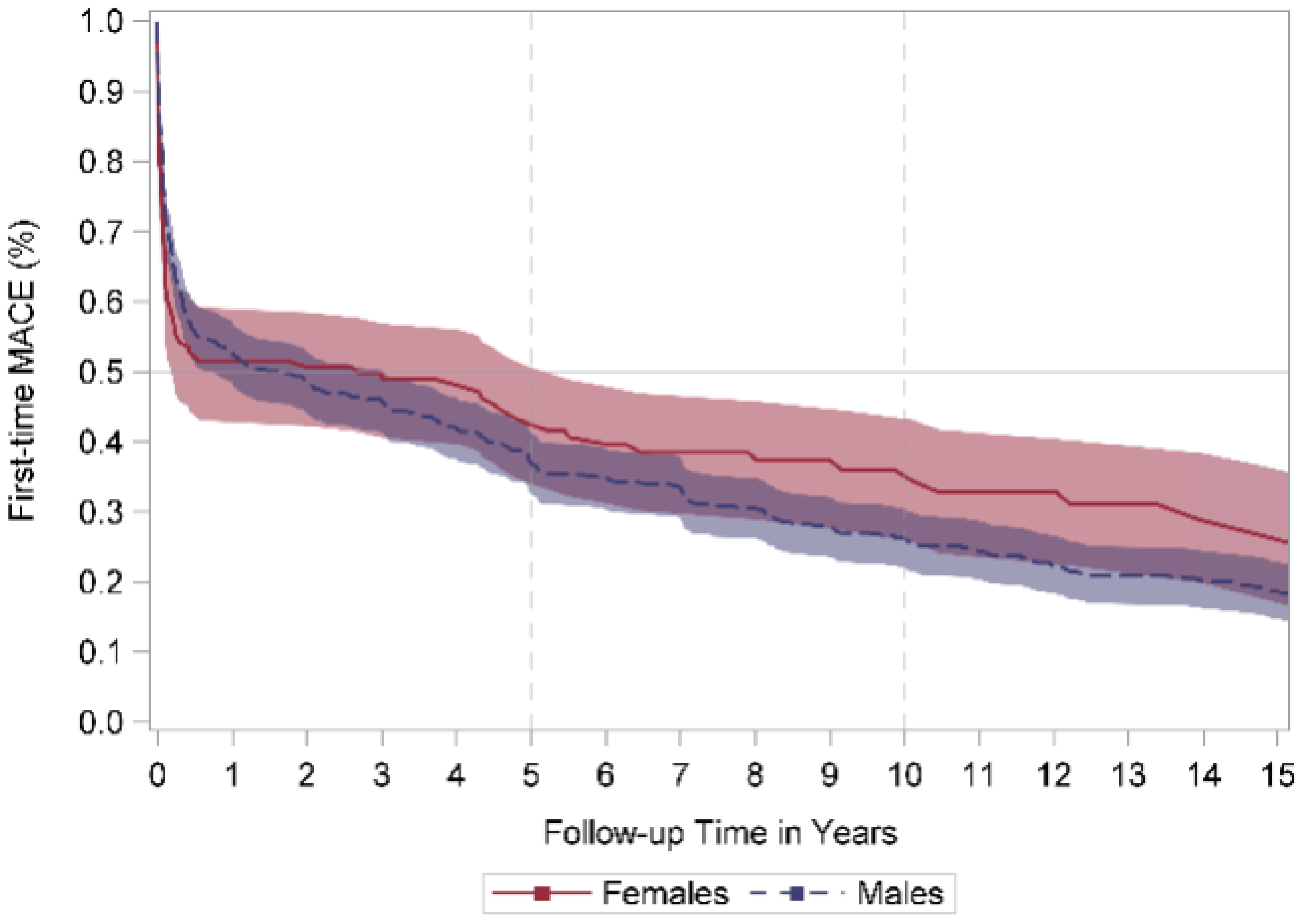

FIGURE S4 Long-term first-time MACE by gender

Conditional first-time MACE in recipients who survived first year after the index date (1-year Post-surgery MACE) stratified by female (red) and male (blue). Due to data protection, recipients were only followed during 15 years after index date.

MACE, Major Adverse Cardiovascular Event (composite of readmission due to heart failure, graft failure, percutaneous coronary intervention, and all-cause mortality).
